# Pulmonary nodules near fissures in hepatocellular carcinoma: assessing their clinical significance

**DOI:** 10.31744/einstein_journal/2025AO1895

**Published:** 2025-11-24

**Authors:** Leonardo Chaves Machado, Gilberto Szarf, Gustavo Borges da Silva Teles, Patrícia Yokoo, Rodrigo Caruso Chate, Eduardo Kaiser Ururahy Nunes Fonseca

**Affiliations:** 1 Hospital Israelita Albert Einstein São Paulo SP Brazil Hospital Israelita Albert Einstein, São Paulo, SP, Brazil.

**Keywords:** Multiple pulmonary nodules, Carcinoma, hepatocellular, Liver neoplasms, Diagnostic imaging, Neoplasm metastasis

## Abstract

**Objective:**

To evaluate whether pulmonary nodules adjacent to fissures, particularly perifissural nodules detected on chest computed tomography in patients with hepatocellular carcinoma, can be reliably classified as benign based on their radiological characteristics.

**Methods:**

This retrospective cohort study included patients diagnosed with hepatocellular carcinoma (LI-RADS 5) between 2018 and 2022 at two reference hospitals. The patients underwent at least two follow-up chest computed tomography scans, with a minimum interval of 1 year, to identify at least one pulmonary nodule adjacent to the fissures. Statistical analyses were performed to assess the nodule classification, growth rates, and their association with metastatic progression.

**Results:**

In total, 55 patients (mean age 62.4±7.7 years) and 92 nodules were analyzed. Perifissural nodules were classified as typical (n=55, 59.8%), atypical (n=17, 18.5%), or non-perifissural nodule (n=20, 21.7%). Only two nodules (2.2%) progressed to metastasis: one non-perifissural nodule and one atypical perifissural nodule with histopathological confirmation. Statistical analysis did not reveal a significant association between perifissural nodule classification and metastatic progression (p=0.155); however, nodules that progressed to metastasis exhibited significantly higher annual growth rates (p<0.001).

**Conclusion:**

Most perifissural nodules in patients with hepatocellular carcinoma showed radiological stability and did not progress to metastasis, indicating a low probability of malignancy. However, given the limited sample size and the number of metastatic events, these findings should be interpreted with caution. Typical perifissural nodules may be considered low risk, whereas atypical perifissural nodule and non-perifissural nodules require closer follow-up. Further studies are required to confirm these preliminary findings.

## In Brief

In patients with hepatocellular carcinoma, typical perifissural nodules are stable with no metastatic potential. Atypical perifissural nodules and non-perifissural nodules are uncommon but occasionally metastatic, suggesting the need for tailored surveillance.

## Highlights

■ Typical perifissural nodules remain radiologically stable with no metastatic potential.
■ Only two nodules progressed to metastasis (one atypical perifissural nodule and one non-perifissural nodule).
■ Atypical and non-perifissural nodules require closer follow-up.
■ Annual growth rate was a strong marker of malignancy.

## Graphical abstract

**Figure:**
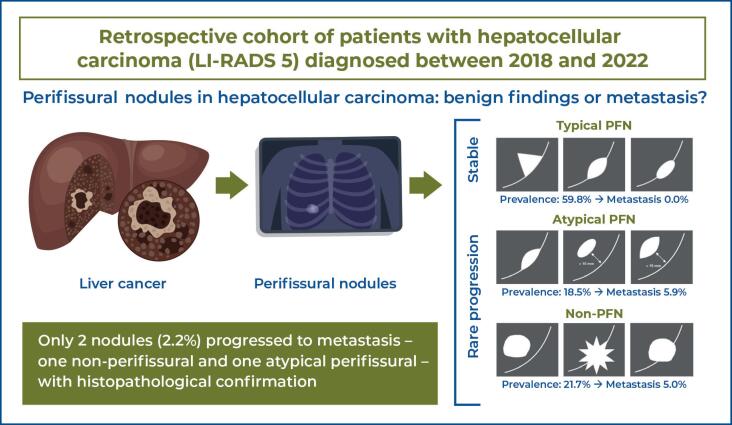

## INTRODUCTION

Primary liver cancer is the sixth most prevalent type of cancer and the fourth leading cause of cancer-related deaths, with an estimated incidence of at least nine cases per 100,000 inhabitants per year and nearly 800,000 deaths in 2018.^(1)^ Hepatocellular carcinoma (HCC) is the most common histological subtype, affecting approximately 75% of patients, whereas intrahepatic cholangiocarcinoma is the second most frequent subtype, accounting for only 12-15% of cases.^(1)^ The primary risk factor for HCC is liver cirrhosis.^(2)^

The literature demonstrates that the lungs are the primary site of distant metastasis from HCC, followed by the abdominal lymph nodes and bones.^(2)^ A study by Katyal et al. reported that more than half of patients with HCC developed pulmonary metastasis,^(3)^ a rate similar to that observed in another retrospective study of 53.8%.^(4)^ The hematogenous route is the main mechanism of dissemination, with the lower lung lobes preferentially affected.^(2)^ The lesions are usually seen as non-calcified pulmonary nodules.^(2)^

In the absence of a known oncological context, there are guidelines for the management of incidental nodules detected on computed tomography (CT), such as the 2017 Fleischner Society^(5)^ consensus and 2015 British Thoracic Society^(6)^ directives. Characteristics, such as nodule composition (solid or subsolid), size, quantity (single or multiple), morphology, and association with other risk factors, determine the individual risk of each nodule and the subsequent management, which may include follow-up at different intervals, additional radiological methods (positron emission tomography (PET)/CT), biopsy, or even no follow-up.^(5)^

**Figure 1:**
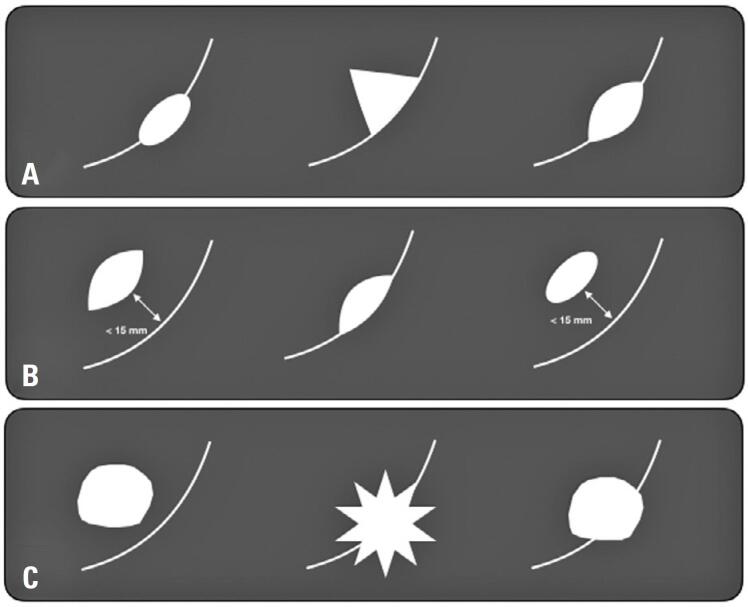
Classification of nodules adjacent to pulmonary fissures. Schematic representation of typical, atypical, and non-perifissural (PFN) nodules. Typical nodules are solid, non-calcified, and exhibit oval, triangular, or lentiform shapes, measuring up to 12mm (A). Atypical nodules may share similar characteristics but differ in their attachment or distance from the fissure (B). Non-PFNs do not meet these criteria (C)

**Figure 2:**
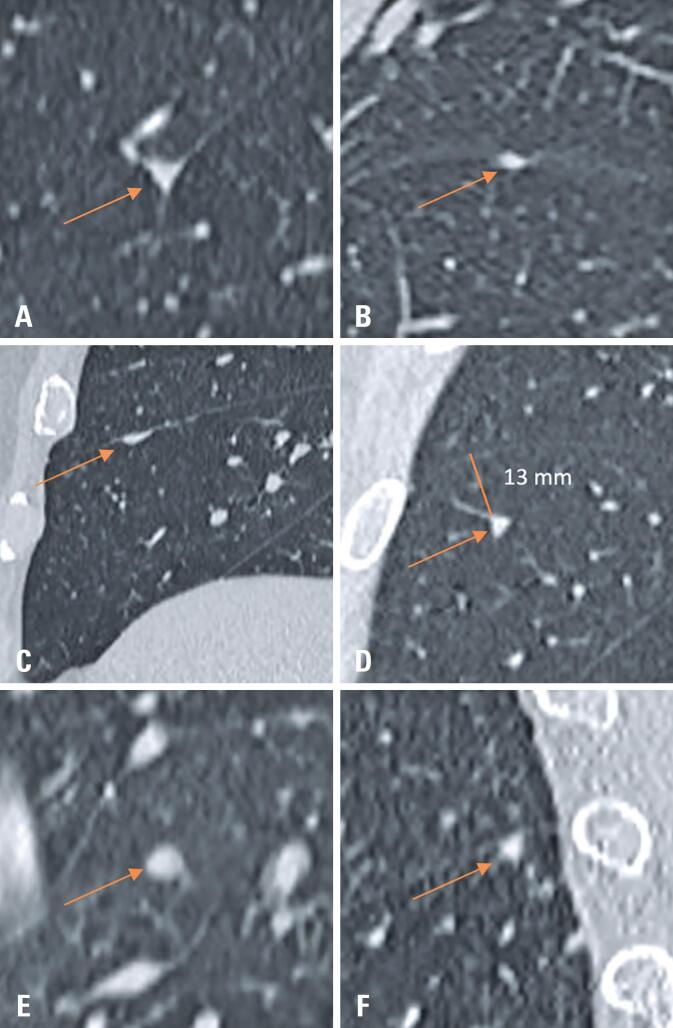
Chest computed tomography examples of the types of nodules adjacent to pulmonary fissures. Chest computed tomography scans illustrating examples of typical perifissural nodules (PFNs) (A, B), atypical PFNs (C, D), and non-PFNs (E, F), identified in the study population. Each panel highlights distinguishing morphological features, including attachment, shape, and proximity to the fissures

In some scenarios, the nodules are located adjacent to or attached to the pulmonary fissures. Among these, a distinct subtype, perifissural nodules (PFNs), which represent intrapulmonary lymph nodes, can typically be differentiated from primary malignant lesions based on their characteristic morphology.^(7)^ They are characterized as solid, non-calcified nodules with well-defined margins and a triangular, oval, or lentiform shape, with a diameter of up to 12mm.^(7)^ Perifissural nodules are classified as "typical" or "atypical." Atypical nodules share the features of typical nodules but differ in their attachment; they are either unattached and located within 15mm of the pleura or fissure, or attached but display a convex shape on one side and a rounded shape on the other.^(8)^ Non-PFNs did not meet these criteria (Figures 1 and 2).

Despite the risk of pulmonary metastasis in patients with HCC,^(2-4)^ no studies have focused on this population regarding the evaluation of pulmonary nodules and the significance of those adjacent to fissures and PFNs, a common finding in the general population. This creates diagnostic uncertainties in oncological management, especially when they demonstrate an increase in size.^(9)^

It is imperative to understand which characteristics of a pulmonary nodule can be used to safely exclude the possibility of secondary involvement, particularly in the context of malignancies with a high propensity for pulmonary metastasis, such as HCC. More specifically, it is desirable to ascertain whether PFNs/intrapulmonary lymph nodes can be deemed benign, even within an oncological context, thereby minimizing unnecessary invasive procedures and healthcare costs.

## OBJECTIVE

To evaluate whether pulmonary nodules adjacent to fissures, particularly perifissural nodules detected on chest computed tomography in patients with hepatocellular carcinoma, can be reliably classified as benign based on their radiological characteristics.

## METHODS

All patients with HCC diagnosed through imaging (CT or magnetic resonance imaging (MRI) with LI-RADS 5 criteria^(10)^) aged 18 years or older, and treated at *Hospital Israelita Albert Einstein* or *Hospital Municipal Gilson de Cássia Marques de Carvalho* between 2018 and 2022, with at least one staging chest CT were included in this study. The present study was approved by the Ethics Committee of the *Hospital Israelita Albert Einstein* (CAAE: 66536122.6.0000.0071; # 5.922.779).

### Inclusion criteria

The inclusion criteria include patients diagnosed with HCC classified as LI-RADS 5, aged 18 years or older, treated at the aforementioned institutions between 2018 and 2022.

### Exclusion criteria

The exclusion criteria included a lack of chest CT follow-up with a minimum interval of 1 year, the absence of pulmonary nodules adjacent to fissures, simultaneous primary neoplasms at other sites, previously identified pulmonary metastases, and imaging artifacts that hindered proper pulmonary evaluation.

### Image evaluation

Chest CT scans were independently evaluated in a random order by two thoracic radiologists, each with over 10 years of experience, who were blinded to the patients’ clinical information. Subsequently, all cases were jointly reviewed by a third thoracic radiologist with more than 30 years of experience to reach a consensus classification for each nodule.

### Statistical analysis

Data were analyzed in an aggregate format with the support of a dedicated statistical department. The variables were classified as either categorical or quantitative to facilitate the application of appropriate statistical tests. The association between nodule classification and metastasis was assessed using the likelihood ratio test. The Mann-Whitney U test was used to compare the maximum and mean annual growth rates between metastatic and non-metastatic nodules. The p-values were calculated for all analyses, with values <0.05 considered statistically significant.

## RESULTS

**Figure 3:**
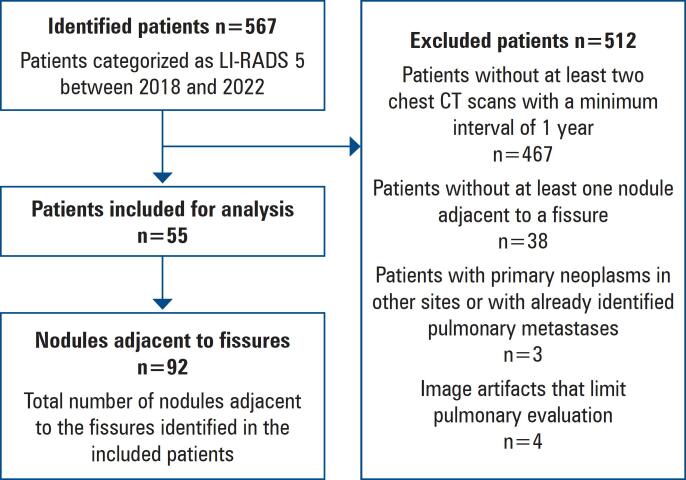
Study population flowchart

The initial patient selection identified 567 individuals with LI-RADS 5 from January 1, 2018, to December 31, 2022. Of these, 512 were excluded based on the following specific criteria: 467 did not have at least two thoracic CT scans performed at an interval of at least 1 year, 38 did not present nodules near pulmonary fissures, three had already been diagnosed with primary neoplasms at another site or had pulmonary metastasis, and four had imaging studies with artifacts that compromised the analysis. Therefore, 55 eligible patients remained, and 92 nodules were evaluated. Figure 3 shows a flowchart of the patient and nodule selection processes.

Of the 567 initially identified patients, the following exclusion criteria were applied: insufficient imaging intervals, lack of nodules near the fissures, primary neoplasms at other sites, previously identified pulmonary metastases, or imaging artifacts. Ultimately, 55 patients with 92 nodules were included in the analysis.

The mean patient age was 62.4 years (±7.7), of whom 89.1% were male. More than half of the patients (50.9%) had undergone some type of local hepatic treatment, such as resection and/or ablation, before the abdominal imaging examination used for study inclusion. The mean follow-up time was 2.8 years (range: 1.2-7.4 years).

**Figure 4:**
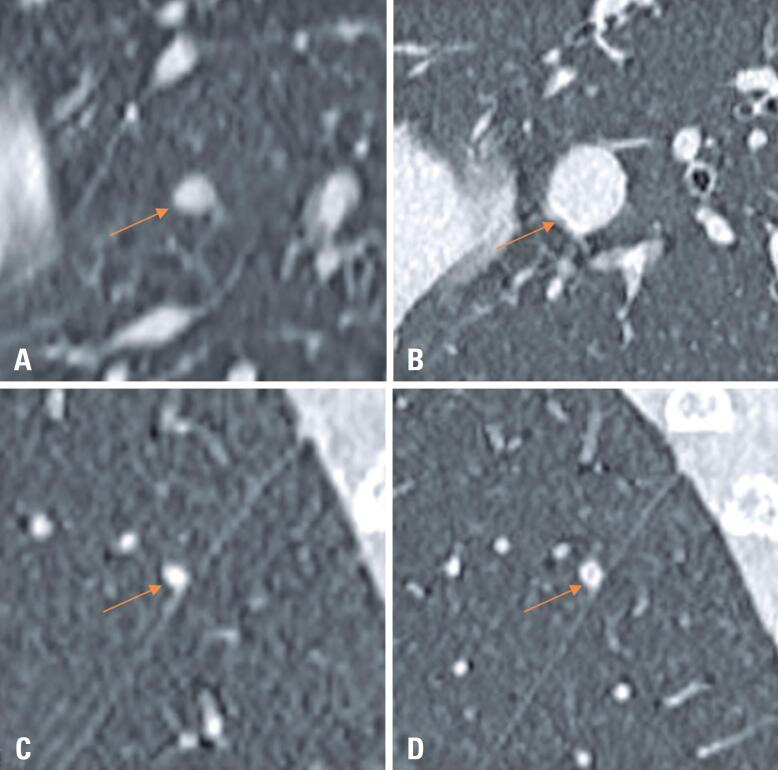
Evolution of nodules progressing as pulmonary metastases. Computed tomography scans demonstrating the progression of nodules to metastatic lesions. Panel (A, B): a non-perifissural nodule showing significant growth over 3 years. Panel (C, D): an atypical perifissural nodule exhibiting slight growth and morphological changes, confirmed as metastatic upon histopathological examination

Classification based on the nodule characteristics revealed 55 typical PFNs (59.8%), 17 atypical PFNs (18.5%), and 20 non-PFNs (21.7%). Regarding the PFN morphology, nearly half exhibited a triangular shape, while the remainder were almost equally distributed between the oval and lentiform shapes. During follow-up, only two of the 92 nodules adjacent to the pulmonary fissures (2.2%) progressed to pulmonary metastasis: one was identified as a non-PFN (radiological diagnosis consistent with secondary involvement), while the other was classified as an atypical PFN, with histopathological confirmation (Table 1 and Figure 4).

**Table 1:** Characteristics of the pulmonary nodules

Variable		n (%)
Classification		
	Typical	55 (59.8)
	Atypical	17 (18.5)
	Non-PFN	20 (21.7)
Shape of PFNs		
	Oval	17 (23.9)
	Lentiform	18 (25)
	Triangular	37 (51.1)
Metastasis		
	No	90 (97.8)
	Yes	2 (2.2)
Total		92 (100)

A likelihood ratio test was performed to assess the relationship between nodule classification and metastasis in the sample of 92 nodules. The test yielded a p-value of 0.155, indicating that the association between nodule classification and presence of metastasis was not statistically significant (Table 2).

**Table 2:** Statistical analysis of the nodules

Variable		Metastasis		Total n (%)	p value
		No n (%)	Yes n (%)		
Classification					0.155
	Typical	55 (61.1)	0 (0.00)	55 (59.8)	
	Atypical	16 (17.8)	1 (50.0)	17 (18.5)	
	Non-PFN	19 (21.1)	1 (50.0)	20 (21.7)	
	Total	90 (100)	2 (100)	92 (100)	

Likelihood Ratio Test.

The analysis of the annual maximum and mean growth rates revealed statistically significant differences between metastatic and non-metastatic nodules (p<0.001 for both variables, Mann-Whitney U test). Metastatic nodules exhibited significantly higher annual growth rates, with a maximum rate of 0.31 mm/year and a mean rate of 0.30 mm/year, compared to non-metastatic nodules, which demonstrated stability (Table 3).

**Table 3:** Analysis of the annual maximum and mean growth rates

Variable	Metastasis	Mean	SD	Median	Minimum	Maximum	n	p value
Annual maximum growth rate (mm/year)	No	–0.01	0.07	0.00	–0.48	0.08	90	<0.001
	Yes	0.31	0.01	0.31	0.31	0.32	2	
	Total	–0.01	0.09	0.00	–0.48	0.32	92	
Annual mean growth rate (mm/year)	No	–0.01	0.07	0.00	–0.48	0.03	90	<0.001
	Yes	0.30	0.02	0.30	0.29	0.31	2	
	Total	–0.01	0.08	0.00	–0.48	0.31	92	

Mann–Whitney Test.

## DISCUSSION

In this study, we aimed to characterize the behavior of nodules adjacent to fissures in patients with HCC, highlighting the differences between the typical PFN, atypical PFN, and non-PFN subtypes. Classification and follow-up of the nodules allowed us to identify specific patterns, contributing to radiological interpretation in an oncological context. Our analysis revealed that only two of the 92 evaluated nodules progressed to pulmonary metastasis, representing 2.2% of the sample, confirming the low rate of malignant transformation.

Previous studies, such as that by Golia Pernicka et al., indicated that 95.2% of PFNs in oncological populations remained stable or decreased in size over an average follow-up of 5.7 years.^(11)^ While our results similarly showed a low transformation rate (2.2%), they differed in the classification of nodules that progressed to metastasis. In Pernicka's study, all nodules that increased in size maintained benign characteristics in subsequent evaluations, whereas in our study, atypical PFNs exhibited growth and transformation, as confirmed by histopathological examination. This discrepancy may be attributed to the specificity of our HCC patient population, which was characterized by a high propensity for hematogenous dissemination.^(3,4)^

A noteworthy study by de Hoop et al. investigated PFNs in patients undergoing lung cancer screening.^(8)^ While they reported the growth of PFNs, no cases of malignancy were identified.^(8)^ In contrast, we documented an atypical PFN that underwent malignant transformation, highlighting the importance of caution when evaluating these lesions in the context of primary malignancies with high metastatic potential. Similarly, Pomerri et al. explored the significance of pulmonary nodules in patients with colorectal cancer and revealed that these nodules often present diagnostic challenges similar to those encountered in our study.^(12)^ Despite addressing distinct primary cancer types, both studies underscore the critical need for a thorough assessment of the radiological features of nodules in oncological contexts with an elevated risk of metastasis.

The morphology and annual growth of the nodules were systematically analyzed. Nodules classified as typical exhibited significant stability, corroborating the findings of Ahn et al., who demonstrated the low aggressiveness of PFNs, even in high-risk populations.^(13)^ However, the progression observed in an atypical PFN in our cohort highlights the importance of considering morphological characteristics and clinical context rather than relying solely on general population patterns.

Additionally, the association between growth rates and malignant potential aligns with findings from studies such as those of Stephens et al., which emphasized that malignant nodules tend to exhibit higher growth rates than benign nodules.^(9)^ In our sample, nodules that progressed to metastasis showed significantly higher annual growth rates (maximum of 0.31 mm/year) compared to other lesions.

Although our results offer a relevant contribution to the characterization of PFNs in patients with HCC, this study has some limitations that should be acknowledged. The sample size was limited, and the number of metastatic events was low, which may have reduced the statistical power to detect significant associations. Additionally, no multivariate analysis was performed to control for potentially confounding clinical or radiological variables, such as tumor burden, disease stage, or prior treatments. These factors may have influenced the outcomes and interpretation of the nodule behavior. Therefore, the results should be interpreted with caution. Further studies with larger sample sizes and more robust statistical approaches are needed to confirm these findings and enhance their generalizability.

## CONCLUSION

This study suggests that most pulmonary nodules adjacent to fissures, particularly those with a typical perifissural morphology, tend to remain radiologically stable and are unlikely to represent metastatic disease in patients with hepatocellular carcinoma. However, owing to the limited number of malignant cases observed and the absence of a multivariate analysis, these findings should be interpreted with caution.

Although the results are consistent with previous literature on non-oncologic populations, the small number of events and the retrospective design restrict the statistical power and generalizability of the conclusions. Atypical perifissural nodules and non-perifissural nodules may require closer monitoring; however, further prospective and multicenter studies with larger sample sizes are necessary to validate these preliminary observations and define standardized follow-up protocols.
